# Cerebrovascular Disorders

**DOI:** 10.1186/s12872-023-03225-8

**Published:** 2023-04-28

**Authors:** Alessandra Granata, Eric L Harshfield, Joseph V Moxon

**Affiliations:** 1 Department of Clinical Neurosciences, University of Cambridge, Cambridge, UK; 2 Heart and Lung Research Institute, University of Cambridge, Cambridge, UK; 3 College of Medicine and Dentistry, James Cook University, Townsville, Australia

## Abstract

Cerebrovascular disorders pose a global health concern. Advances in basic and clinical research, including induced pluripotent stem cell models and multi-omic approaches, have improved our understanding and management of these disorders. However, gaps in our knowledge remain. *BMC Cardiovascular Disorders invites authors to submit articles investigating* what drives and affects Cerebrovascular disorders to improve patient care.

Cerebrovascular disorders comprise a range of distinct pathologies, including ischaemic and haemorrhagic stroke, transient ischaemic attack, and other intracranial vascular disorders (e.g. vascular malformations and unruptured aneurysms), and significantly increase the risk of vascular dementia. Cerebrovascular disorders represent a significant medical and economic challenge anticipated to rise due to the increasing age of the global population [1]. The past decade has brought considerable progress in diagnostic and therapeutic options, but more needs to be done to improve the life of patients. Advances in basic science techniques and medical technologies hold great potential to improve the management of cerebrovascular disorders in the coming decades; however, rigorously designed translational research programs are needed to bring novel discoveries to the clinic. *BMC Cardiovascular Disorders* welcomes submissions to its new “Cerebrovascular Disorders” collection to help further our understanding of such conditions and open new avenues for risk stratification and therapeutic intervention.

Recent progress in state-of-art-technologies, such as patient-derived human induced pluripotent stem cells (iPSCs) models, 3D technology, multi-omics and high-throughput screening, have opened the door to a better understanding of the mechanisms responsible for cerebrovascular disorders as well as the potential for future therapies. Recently, Xin-Yao Sun et al. [2] have developed vascularised brain organoids by combining brain organoids (mini brains) with a vascular network with features resembling the blood-brain barrier as well as with microglia cells. For the first time, these vascularised brain organoids offer the opportunity to study the interactions between the neuronal and non-neuronal components during disease progression as well as a platform for in vitro drug screening. The limitation of this work is that the vascular network lacks active blood flow, which could be overcome by combining organoid culture with microfluidic techniques. iPSC-derived models that replicate disease-relevant phenotypes could be valuable for high-throughput screenings. However, there are challenges to adopting these models for drug discovery, including heterogeneity, maturity, reproducibility, and scalability. We have successfully adopted an iPSC-derived vascular smooth muscle cells model for *HDAC9* large-vessel stroke risk to screen for histone deacetylase (HDAC) small molecule inhibitors [3]. Our iPSCs model provides a robust screening system. However, the model will require scaling up into 384 or 1536-well plates by adopting liquid handling and robotic manipulation to make it useful for drug screening approaches.

Multi-omics approaches can also help expand our knowledge of cerebrovascular disorders by identifying key molecules to further explore as biomarkers or therapeutic targets. An example is the integrative transcriptomic and proteomic analysis adopted by Ramiro et al. [4] to identify protein and gene expression changes in the post-ischaemic human brain. This approach allowed them to identify dysregulated molecules which were validated in blood samples and presented as potential candidates to become blood biomarkers for both the diagnosis and prognosis of stroke. However, this study used brain samples from deceased patients, which may be affected by protein degradation. Moreover, the affected brain regions differed among patients, influencing protein and gene expressions. The authors used the contralateral hemisphere as a respective individual control to account for this variability. Larger multi-omics studies in various patient populations would be beneficial to further understand and identify the most promising biomarkers.

A challenge with multi-omics data is that there is often extensive pleiotropy, making it difficult to identify which associations are likely to be truly causal. Borges et al. [5] conducted a genome-wide association study of circulating polyunsaturated fatty acids (PUFAs) in nearly 115,000 individuals and used two-sample Mendelian randomization. This statistical technique uses genetic variants as instrumental variables to assess causality in an approach akin to a randomized trial [6] to investigate the link between cerebrovascular and other cardiovascular diseases. They did not find evidence that PUFAs are protective against stroke and other outcomes, largely due to bias caused by horizontal pleiotropy. More advanced statistical techniques are needed to help account for horizontal pleiotropy and disentangle the causal effects across different metabolic pathways.

The above examples highlight the potential for ever-evolving research approaches to improve our understanding of cerebrovascular conditions. In this light, it is important to remain mindful that the ultimate goal of our collective work is to improve the management and welfare of patients and their families. Translating novel research findings into impactful outcomes for consumers is, however, notoriously challenging. Heterogeneity in the design of independent studies (e.g. choice of model system, technologies employed and variations in inclusion and exclusion criteria used to recruit participants) and reporting of outcomes can complicate the generalisability of findings and subsequent uptake. In recognition of this, there is increasing emphasis on the adoption of standardized research approaches to improve the translational potential of pre-clinical (e.g. [7, 8]) and clinically-focused investigations (e.g. [9, 10]). The consumer perspective is also key, and multi-disciplinary studies aiming to address research priorities highlighted by patient advocacy groups and clinicians are likely to deliver more impactful outcomes (e.g. [11]). We welcome submissions with this clear translational intent to this special collection.

We look forward to the “Cerebrovascular Disorders” article collection bringing together basic and clinical research to develop our understanding of what drives and affects such conditions to improve patient care.

## Data Availability

Not applicable.

## List of abbreviations

HDAC: histone deacetylase
iPSC: induced pluripotent stem cells
PUFAs: polyunsaturated fatty acids

## References

[1] Feigin VL, Stark BA, Johnson CO (2021). Global, regional, and national burden of stroke and its risk factors, 1990–2019: a systematic analysis for the global burden of Disease Study 2019. Lancet Neurol.

[2] Sun XY, Ju XC, Li Y, Zeng PM, Wu J, Zhou YY (2022). Generation of Vascularized Brain Organoids to study neurovascular interactions. eLife.

[3] Granata A, Kasioulis I, Serrano F, Cooper JD, Traylor M, Sinha S (2022). The histone deacetylase 9 stroke-risk variant promotes apoptosis and inflammation in a human iPSC-Derived smooth muscle cells model. Front Cardiovasc Med.

[4] Ramiro L, García-Berrocoso T, Briansó F (2021). Integrative multi-omics analysis to Characterize Human Brain Ischemia. Mol Neurobiol.

[5] Borges MC, Haycock PC, Zheng J (2022). Role of circulating polyunsaturated fatty acids on cardiovascular diseases risk: analysis using mendelian randomization and fatty acid genetic association data from over 114,000 UK Biobank participants. BMC Med.

[6] Sanderson E, Glymour MM, Holmes MV (2022). Mendelian randomization. Nat Rev Methods Primers.

[7] Fisher M, Feuerstein G, Howells DW, Hurn PD, Kent TA, Savitz SI et al. Stroke 2009 Vol. 40 Issue 6 Pages 2244-50 10.1161/STROKEAHA.108.541128 PMC2888275 19246690

[8] Percie du N, Sert V, Hurst A, Ahluwalia S, Alam MT, Avey M, Baker et al. BMJ Open Sci 2020 Vol. 4 Issue 1 Pages e100115 10.1136/bmjos-2020-100115 PMC7610906 34095516

[9] Bossuyt PM, Reitsma JB, Bruns DE, Gatsonis CA, Glasziou PP, Irwig L (2015). Stard 2015: an updated list of essential items for reporting diagnostic accuracy studies. BMJ.

[10] Ghaferi AA, Schwartz TA, Pawlik TM (2021). STROBE reporting guidelines for Observational Studies. JAMA Surg.

[11] Hill G (2022). Lancet Neurol.

